# Proton pump inhibitors stabilize the expression of PD‐L1 on cell membrane depending on the phosphorylation of GSK3β

**DOI:** 10.1002/cam4.7083

**Published:** 2024-05-16

**Authors:** Long Gao, Yuan Liu, Jiaying Liu, Jiali Li, Haotian Li, Yanyan Liu, Fang Meng, Xiaohong Du, Yufeng Gao, Jiabin Li, F. Xiao‐Feng Qin

**Affiliations:** ^1^ Department of Infectious Disease The First Affiliated Hospital of Anhui Medical University Hefei China; ^2^ Market Supervision Administration of Xiangcheng District Suzhou China; ^3^ National Key Laboratory of Immunity and Inflammation Suzhou Institute of Systems Medicine, Chinese Academy of Medical Sciences and Peking Union Medical College Suzhou Jiangsu China; ^4^ Key Laboratory of Synthetic Biology Regulatory Elements Suzhou Institute of Systems Medicine, Chinese Academy of Medical Sciences and Peking Union Medical College Suzhou Jiangsu China; ^5^ Suzhou Hospital, Affiliated Hospital of Medical School Nanjing University Suzhou China

**Keywords:** GSK3β, immune checkpoint inhibitors, PD‐L1, proton pump inhibitors

## Abstract

**Background:**

Preclinical and clinical evidence indicates that proton pump inhibitors (PPIs) may indirectly diminish the microbiome diversity, thereby reducing the effectiveness of immune checkpoint inhibitors (ICIs). Conversely, recent publications have shown that PPIs could potentially enhance the response to ICIs. The precise mechanism through which PPIs modulate the ICIs remains unclear. In this study, we discovered a novel molecular function of PPIs in regulating immune invasion, specifically through inducing PD‐L1 translocation in various tumor cells.

**Methods:**

C57BL/6 mice subcutaneous transplantation model is used to verify the potential efficacy of PPIs and PD‐L1 antibody. Western blotting analysis and phosphorylated chip are used to verify the alteration of PD‐L1‐related pathways after being treated with PPIs. The related gene expression is performed by qRT‐PCR and luciferase reporter analysis. We also collected 60 clinical patients diagnosed with esophageal cancer or reflux esophagitis and then detected the expression of PD‐L1 in the tissue samples by immunohistochemistry.

**Results:**

We observed that the IC_50_ of tumor cells in response to PPIs was significantly higher than that of normal epithelial cells. PPIs significantly increased the expression of PD‐L1 on cell membrane at clinically relevant concentrations. Furthermore, pre‐treatment with PPIs appeared to synergize the efficiency of anti‐PD‐L1 antibodies in mouse models. However, PPI administration did not alter the transcription or total protein level of PD‐L1 in multiple tumor cells. Using a phosphorylated protein chip, we identified that PPIs enhanced the phosphorylation of GSK3β, then leading to PD‐L1 protein translocation to the cell membranes. The capacity of PPIs to upregulate PD‐L1 was negated following GSK3β knockout. Furthermore, our clinical data showed that the PPIs use resulted in increased PD‐L1 expression in esophageal cancer patients.

**Conclusion:**

We mainly address a significant and novel mechanism that the usage of PPIs could directly induce the expression of PD‐L1 by inducing GSK3β phosphorylation and facilitate primary tumor progression and metastasis.

## Graphical abstract

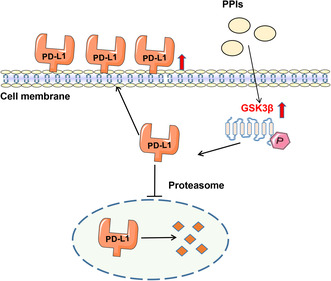

## 1 INTRODUCTION

Immune checkpoint inhibitors (ICIs) targeting CTLA‐4 and PD‐1/PD‐L1 antibodies have already made a greatly breakthrough to improve the outcomes of multiply malignancies.^1^ The application of PD‐1/PD‐L1 targeted immunotherapy has proven successful in treating a range of tumors. But choosing appropriate strategy and timing for the application of ICIs continues to pose significant challenges and uncertainty. Recently, plenty of clinical studies have reported that more than 20% of patients treated with PD‐1/PD‐L1 antibodies experience multiple immune‐related adverse events (irAEs), including rash, fatigue, loss of appetite, peptic ulcers, bloating, and nausea.^2^, ^3^ Such irAEs may lead to surgical ineligibility, postponement of surgical interventions, and a heightened risk of postoperative complication. Some irAEs, such as hemophagocytic lymph histiocytosis, can be systemic and life‐threatening.^4^, ^5^ During clinical management, various drugs, including glucocorticoids, diphenhydramine, loperamide, and proton pump inhibitors (PPIs) are frequently utilized to mitigate these irAEs. Current understanding regarding the contribution of PPIs on the therapeutic effectiveness of PD‐1/PD‐L1 antibody remains a subject of debate, with no definitive evidence to ascertain whether PPIs could influence the effectiveness of immune checkpoints by modulating PD‐L1 expression.^2^, ^3^, ^6^

PPIs are functioned as the inhibitors of H^+^‐K^+^‐ATP proton pump, predominantly inhibiting the secretion of H^+^ from gastric parietal cells into the gastric lumen and consequently up‐regulating gastric pH, thus serving the purpose of treating gastric related diseases.^7^ PPIs are extensively utilized not only in the treatment of peptic ulcer,^8^ but also in preventing gastrointestinal acid abnormalities such as gastrointestinal bleeding, dyspepsia, nausea, and acid reflux during tumor immunotherapy.^2^, ^8^, ^9^, ^10^ However, several clinical studies focusing on non‐small cell lung cancer,^11^, ^12^ colon cancer^13^ and melanoma,^12^ and progression‐free survival in patients receiving PD‐1/PD‐L1 antibodies. PPIs are hypothesized to facilitate the proliferation of potentially genotoxic bacteria by diminishing the diversity and balance of gut microbiota, thereby impairing the immune microenvironment and reducing immune checkpoint antibodies effectiveness, potentially accelerating tumor progression.^14^, ^15^ Conversely, other studies suggests that PPIs do not impact overall survival and progression‐free survival in patients treated with PD‐1/PD‐L1 ICIs for non‐small cell lung cancer and advanced hepatocellular carcinoma.^16^, ^17^ Additionally, PPIs administration appears to synergistically enhance the efficacy of ICIs in melanoma, yet it may have a detrimental effect in non‐small cell lung cancer patients.^18^ The inconsistent outcomes of PPIs on the efficacy of immune checkpoint antibodies could be attributed to variations in patient selection criteria, duration of follow‐up and sample size, population characteristics, drug dosage, and treatment regimen. Nevertheless, the exact influence of PPIs on the efficacy of PD‐1/PD‐L1 ICIs warrants further investigation..

Upregulating PD‐L1 expression in tumor cells constitutes an important mechanism influencing the response to ICIs and facilitating immune escape.^1^ Typically, PD‐L1 expression is modulated by a multitude of factors, encompassing inflammatory stimuli and oncogenic pathways, at transcriptional, posttranscriptional, and posttranslational levels within the tumor microenvironment (TME).^19^ Posttranslational modifications including ubiquitination, phosphorylation, glycosylation, palmitoylation, and SUMOylation, are instrumental in controlling protein stability, activation, localization, and degradation.^20^ Posttranslational modifications such as phosphorylation, glycosylation, and ubiquitination are known to contribute to the establishment of a tumor immunosuppressive microenvironment through the regulation of PD‐L1 protein stability. Nonetheless, the precise mechanism through which PPIs modulate the PD‐1/PD‐L1 axis via these modifications has yet to be fully elucidated.^1^, ^21^, ^22^

Various cancers types express high levels of PD‐L1, leveraging the PD‐L1/PD‐1 signaling pathway to circumvent T cell‐mediated immunity.^23^ Consequently, elucidating the risk of PPIs induced immunosuppression presents a promising therapeutic avenue for selectively targeting tumors. We has demonstrated that PPIs promote PD‐L1 translocation in an array of cancer cells in vitro, and the concurrent administration of PD‐L1 inhibitors and PPIs in vivo appears to synergistically impede the progression of lung and colon cancers. PPIs increase the translocation of PD‐L1 onto tumor cell membrane by inducing GSK3β phosphorylation. In addition, immunohistochemical analysis of clinical samples from esophageal cancer patients revealed that PD‐L1 expression levels were significantly elevated in those receiving PPIs compared to the control group.

## 2 MATERIALS AND METHODS

### 2.1 Cell culture and transfection

LLC and H441 (lung adenocarcinoma cell lines), B16 (mouse melanoma cancer cell lines), AKR (esophageal carcinoma cell lines), MC38 (murine colon cancer cell lines), and 293 T (human embryonic kidney 293 T cells) were cultured in DMEM, while LLC cells were maintained in RPMI‐1640. All tumor cells were obtained from American Type Culture Collection. Selected small molecule drugs or inhibitors were administered to the tumor cells to elucidate the biofunctional changes in cells (seen in Table 1), with DMSO or PBS serving as controls. Generation of GSK3β KO and STAT1 KO cells were previously generated by CRISPR‐Cas9 technology. Optimal GSK3β sgRNA targeted sequences were designed using the Chopchop tool and then colony into Lenti‐CRISPR‐V2 or pX458‐CRISPR plasmid according to the manufacturer's recommended protocol (targeted sequences were seen in Table 2).

**TABLE 1:** Selected drugs or small molecule inhibitors for our research.

Name	Resource	Identifier	Concentration
Erlotinib	MCE	HY‐50896	50, 100 nM
AR‐A014418	MCE	HY‐10512	100, 200 nM
ERK1/2 inhibitor 1	MCE	HY‐112287	25, 50 nM
AV412	MCE	HY‐10346	20, 50 nM
PD‐L1	Roche	Atezolizumab	10, 20 mg/kg
Omeprazole	Sigma‐Aldrich	73590‐58‐6	50, 200, 100, 200 μM, 20 mM/kg
Lansoprazole	Sigma‐Aldrich	L8533	100, 200 μM
Esomeprazole	Sigma‐Aldrich	Y0001660	50, 100, 200 μM, 20 mg/kg
Pantoprazole	Sigma‐Aldrich	Y0000835	100, 200 μM
DMSO	Sigma‐Aldrich	D8418	Appropriate
PBS	Sangon biotech	E607008	Appropriate
hIFN‐γ	R&D Systems	CAA31639	10, 20 ng/mL
hIFN‐β	R&D Systems	P01574	10, 20 ng/mL
mIFN‐γ	R&D Systems	NP_032363	10, 20 ng/mL
mIFN‐β	R&D Systems	P01575	10, 20 ng/mL
KCL	Sangon biotech	A610440	5, 10 mM
NaCl	Sangon biotech	A610476	5, 10 mM

**TABLE 2:** Targeted sgRNA sequences for our research.

Name	Sequence
Mouse‐sgGSK3β‐1	GTCTCGGTCGCCCCGACATGA
Mouse‐sgGSK3β‐2	GACTGTAACATAGTCCGACTG
Human‐sgGSK3β‐1	GCGGCTTGCAGCTCTCCGCAA
Human‐sgGSK3β‐2	GTTTGGCTCGACTATAGTGTC
Mouse‐sgAhR‐1	GCTGAAGGAATTAAGTCAAAT
Mouse‐sgAhR‐2	GCGCTGAAGGAATTAAGTCAA
Human‐sgAhR‐1	GGTAAAGCCAATCCCAGCTGA
Human‐sgAhR‐2	GTCAAGTCAAATCCTTCCAAG

### 2.2 Animal studies

LLC and MC38 cells were subcutaneously injected into the wild‐type (WT) C57BL/6 (*n* = 5–6, purchased from the Model Animal Research Center of Nanjing University (Nanjing, China)) at 2 × 10^5^ per mouse. Tumor growth was measured once every 2 days (volume = (length × width × width)/2). All mice used in the experiment were female, 18–20 g and 6–8 weeks old. All groups would be sacrificed and the tumor weight also be recorded. All animal experiments were agreed with animal care, handling, and treatment according to the guidelines approved by the Institutional Animal Care and Use Committee of Suzhou Institute of Systems Medicine (ISM‐IACUC‐0003‐R).

### 2.3 Real‐time quantitative PCR assays

All tumor cells were seeded at a density of 2 × 10^5^ cells/well in 6‐well plates for 24 h, and subsequently treated with control or PPIs for another 12 h or 24 h, and finally collect the cells for further analysis. Total RNA were extracted by Trizol kit according to the manufacturer instructions and the using Takara‐PCR‐kit to reverse transcripted‐RNA into cDNA solution and then all samples stored at −20°C for further analysis. Related genes expression was evaluated by RT‐quantitative PCR. Quantitative PCR was designed by gene‐specific primers and GAPDH as reference gene (seen in Table 3). Samples were then analyzed for mRNA expression via qRT‐PCR by using the Light Cycler 480 (Roche) instrument and the relative expression of mRNA was normalized to the GAPDH.

**TABLE 3:** Designed gene‐specific primers for qPCR.

Name	Sequence
Human GAPDH‐F	GGCATGGACTGTGGTCATGAG
Human GAPDH‐R	TGCACCACCAACTGCTTAGC
Mouse β‐Actin‐F	GGGAAATCGTGCGTGAC
Mouse β‐Actin‐R	AGGCTGGAAAAGAGCCT
Human PD‐L1‐F	GCTGCACTAATTGTCTATTGGG
Human PD‐L1‐R	CACAGTAATTCGCTTGTAGTCG
Mouse PD‐L1‐F	TGAGCAAGTGATTCAGTTTGTG
Mouse PD‐L1‐R	CATTTCCCTTCAAAAGCTGGTC

### 2.4 Western blotting assays

All tumor cells were seeded at a density of 2 × 10^5^ cells/well into 6‐well plates for 24 h and subsequently treated with control or PPIs for another 12 h or 24 h, and finally collect the cells for further detection. Cells were lysed in RIPA lysis buffer and denatured in SDS loading buffer for total proteins. After denaturation, equal protein was separated by 10% SDS‐PAGE gel and transferred onto PVDF. The membranes were blocked with TBST adding 5% nonfat milk and incubated with primary antibodies (seen in Table 4). Secondary antibodies were incubated for 1 h at room temperature, and visualized by the ECL substrate at Chemiluminescence Instrument (BioRad). The detection of PD‐L1 related phospho‐antibody protein chips from H441 cell lines, omeprazole (OME), and non‐OME protein lysates were carried out according to the instructions of the Human Phospho‐Kinase Array Kit (R&D Systems, Cat. ARY003B).

**TABLE 4:** Commercial antibodies for Western blotting.

Name	Resource	Identifier	Concentration
PD‐L1	CST	13684	1:1500
APC‐PD‐L1	eBioscience	47‐5983‐42	1:1000
ERK1/2	Santa Cruz	sc‐93	1:1500
pERK202	Santa Cruz	sc‐101760	1:1200
GSK3β	Proteintech	67329‐1‐Ig	1:1000
GSK3β‐Ser9	Proteintech	67558‐1‐Ig	1:1000
STAT3	CST	9139	1:2000
pSTAT3‐Tyr705	CST	9145	1:1500
V‐ATP ase β1	Thermo Fisher Scientific	PA5‐75580	1:1000
GAPDH	Proteintech	60004‐1‐Ig	1:3000

### 2.5 Cell viability assays

CCK‐8 kits were used to estimate cell proliferation assays. Tumor cells (3000/well) were seeded in 96‐well plates overnight then administrated with different concentration of control, OME, and lansoprazole (LAN) for 24 h or 48 h (seen Table 1). And absorption at 450 nm was detected with a Synergy plate reader (BioTek). The data were representative of three independent experiments.

### 2.6 Immunohistochemistry assays and clinical data collection

IHC staining was described by our previously study in detail.^6^ In briefly, esophageal tissues were obtained from pathology department of the First Affiliated Hospital of the Chengdu Medical College and dissect into 3 μm sections. Tissue slices underwent dewaxing, rehydration, antigen retrieval, blocking, and then incubated with PD‐L1 (1:50) primary antibodies overnight at 4°C. After washed with PBS, slices were incubated with HRP‐related secondary antibody, washed and then developed with DAB. Finally, slices were dehydrated and mounted with coverslips. Pictures were obtained using a Nikon microscope camera and NIS‐Elements software and with a digital whole‐slide scanner (Leica, SCN400F). All esophageal tissue samples were agreed with patients informed consent, usage, and treatment according to the guidelines approved by the Institutional Ethics Committee of the First Affiliated Hospital of the Chengdu Medical College (CDMC‐EC‐0016‐H).

### 2.7 Quantification and statistical analysis

All data are presented as mean and error bars represent standard error of mean (SEM) from at least two or three biological replicates. ANOVA One‐way test, Two‐way test and Chi square test were calculated for statistical analyses. For survival analysis, the data were plotted and compared using the log‐rank test. Pearson's correlation analysis was used to assess the correlation between two genes. All statistical analysis was done using GraphPad Prism (v.6.0).

## 3 RESULTS

### 3.1 PPIs significantly up‐regulate the PD‐L1 membrane expression in tumor cells

**FIGURE 1:**
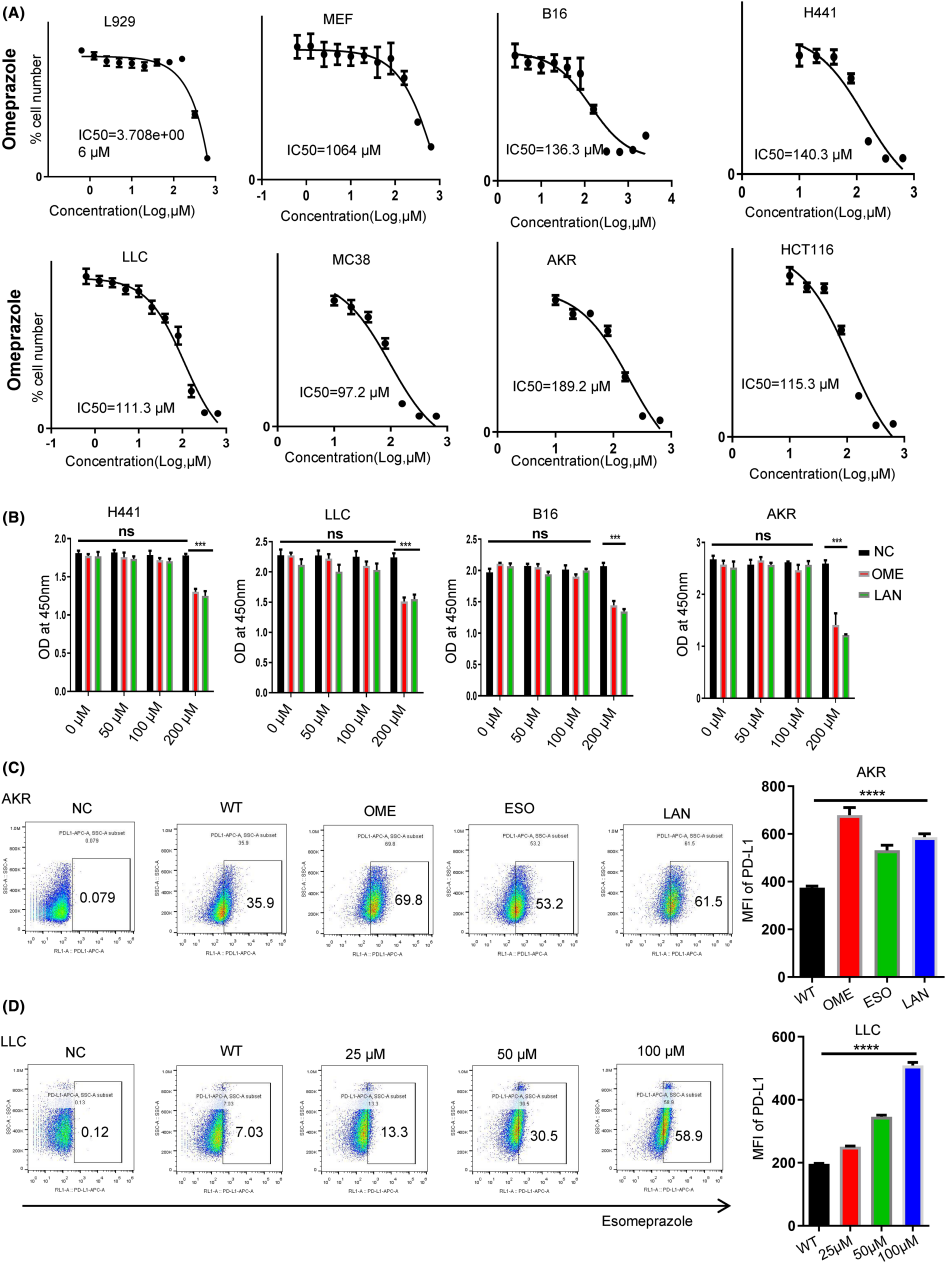
PPIs significantly up‐regulated the PD‐L1 membrane expression in tumor cells. (A) The IC_50_ of multiply tumor cells (B16, H441, LLC, AKR, MC38, and HCT116) and normal endothelial fibroblasts cells (L929 and MEF) were performed after treated with Omeprazole for 12 h. (B) B16, H441, LLC, and AKR cells were seeded at a density of 3000 cells/well into 96‐well plates, subsequently pre‐treated with omeprazole (OME) or lansoprazole (LAN) and for 48 h and then cell viability was determined using a CCK‐8 assay, and the results were shown as the OD value to represent the relative proliferation ability of the cells (*n* = 3). (C) Representative FACS plots showing the relative PD‐L1 expression in AKR cells treated with different type of PPIs (*n* = 3). (D) Representative FACS plots showing the relative PD‐L1 expression in LLC cells treated with the different concentration of esomeprazole for 24 h (ESO) (*n* = 3). *p ≤ 0.05, **p ≤ 0.01, ***p ≤ 0.001, ****p ≤ 0.0001.

Upon administrating multiply tumor cells and two normal endothelial fibroblasts cells with OME, esomeprazole (ESO), pantoprazole (PAN), and LAN, we noted that twe noted that the IC50 values for normal cells in response to PPIs were significantly higher than those for malignant cells (Figure 1A). Moreover, as depicted in Figure 1B, moderate concentrations of PPIs did not impact the cell proliferation, suggesting that clinically reasonable concentrations of PPIs might not have a pronounced cytotoxic effect on normal epithelial cells or cancer cells. Subsequently, we attempted to explore the influence of PPIs on the PD‐L1 expression. To our surprise, multiply tumor cell lines (AKR, H441, MC38, B16, and LLC) amplified the translocation of PD‐L1 on cell member after treated with OME, LAN, and ESO (Figure 1C; Figure S1A). Further investigations using exogenous application of varied concentrations of ESO also showed an enhancement in PD‐L1 membrane expression in tumor cells (Figure 1D; Figure S1B). In order to visualize the subcellular localization of PD‐L1, we further illustrated that OME treatment certainly promoted the PD‐L1 trans‐locate onto cell membrane by immunofluorescence (Figure S4A). These results showed that PPIs promoted PD‐L1 translocation onto tumor cell membrane.

### 3.2 PPIs synergistically enhance the efficacy of PD‐L1 antibody

**FIGURE 2:**
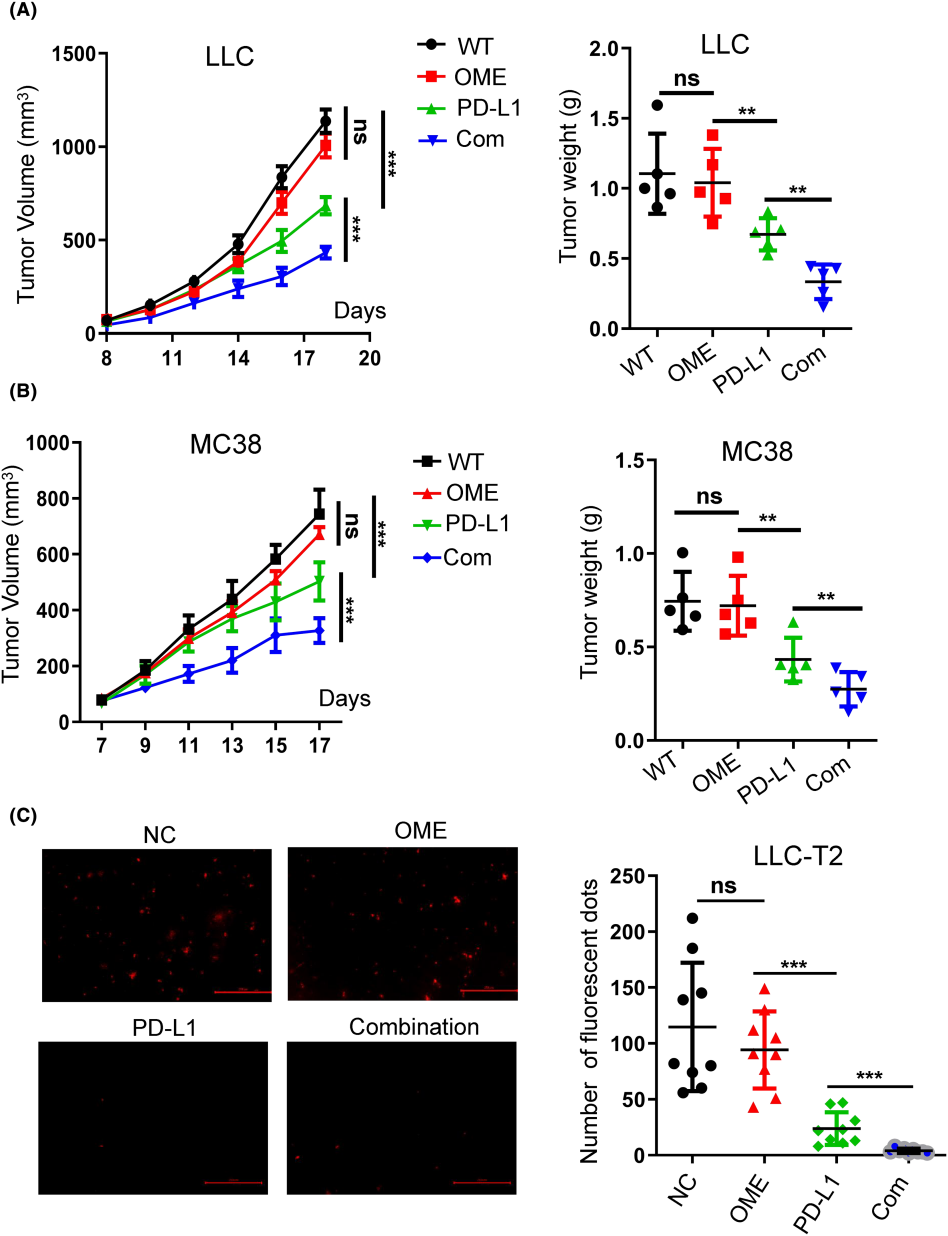
PPIs synergistic enhanced the efficacy of PD‐L1 antibody. (A, B) LLC cells (2 × 10^5^ per mice) (A) or MC38 cells (3.0 × 10^6^ per mice) (B) were separately implanted into C57BL/6 mouse (left). Tumors were measured every 2 days at Day 7, and then omeprazole (20 mg/kg), PD‐L1 antibody (10 mg/kg), the combination of the above drugs, or PBS control was injected into mice (intraperitoneal) twice a week for 2 weeks beginning on the ninth day after LLC tumor cells were subcutaneously implanted (left). The tumor growth curve was shown with tumor sizes (*n* = 5–6). Mice were sacrificed at Day 18 after injection. The primary tumor mass is shown on the right (right). (C) LLC‐T2 cells (2 × 10^5^ per mice) were separately injected into tail vein of C57BL/6 mouse (left), and then omeprazole (20 mg/kg), PD‐L1 antibody (10 mg/kg), the combination of the above drugs, or PBS control was injected into mice (intraperitoneal) twice a week for 2 weeks beginning on the ninth day after LLC tumor cells injected. Number of spontaneous lung metastases in LLC‐T2 tumor‐bearing immunocompetent C57BL/6 mouse was shown. The visual numbers were randomly chosen in each group (*n* = 3), calculated by image J software. *p ≤ 0.05, **p ≤ 0.01, ***p ≤ 0.001, ****p ≤ 0.0001.

Base on the above observations, we attempted to investigate the therapeutical affection of PPIs on anti‐PD‐1/PD‐L1 antibodies using MC38 and LLC tumor cell subcutaneous transplantation models (based on immunocompetent C57BL/6 mouse). Treatment with OME alone did not show any inhibitory effect on tumor growth and tumor weight compared to the control group. However, in combination, OME appeared to synergistically boost the antitumor efficacy of PD‐L1 antibodies (Figure 2A,B). Parallel findings were obtained using ESO in a subcutaneous model of LLC tumor cells, where ESO in combination with PD‐L1 antibody illustrated a more pronounced inhibitory of tumor growth (Figure S2A). To further clarify the role of PPIs on tumor progression, we also transfected a red fluorescent protein into LLC cells (designated as LLC‐T2), enabling visualization of tumor metastasis in vivo. No significant difference of tumor growth was observed between LLC‐T2 and LLC in the mice model (Figure S2B). We found that combined PD‐L1 with OME groups reduced the potential for tumor lung metastasis more effectively than that of control group (Figure 2; Figure S2C).

### 3.3 PPIs do not regulate PD‐L1 mRNA transcription and total protein level

**FIGURE 3:**
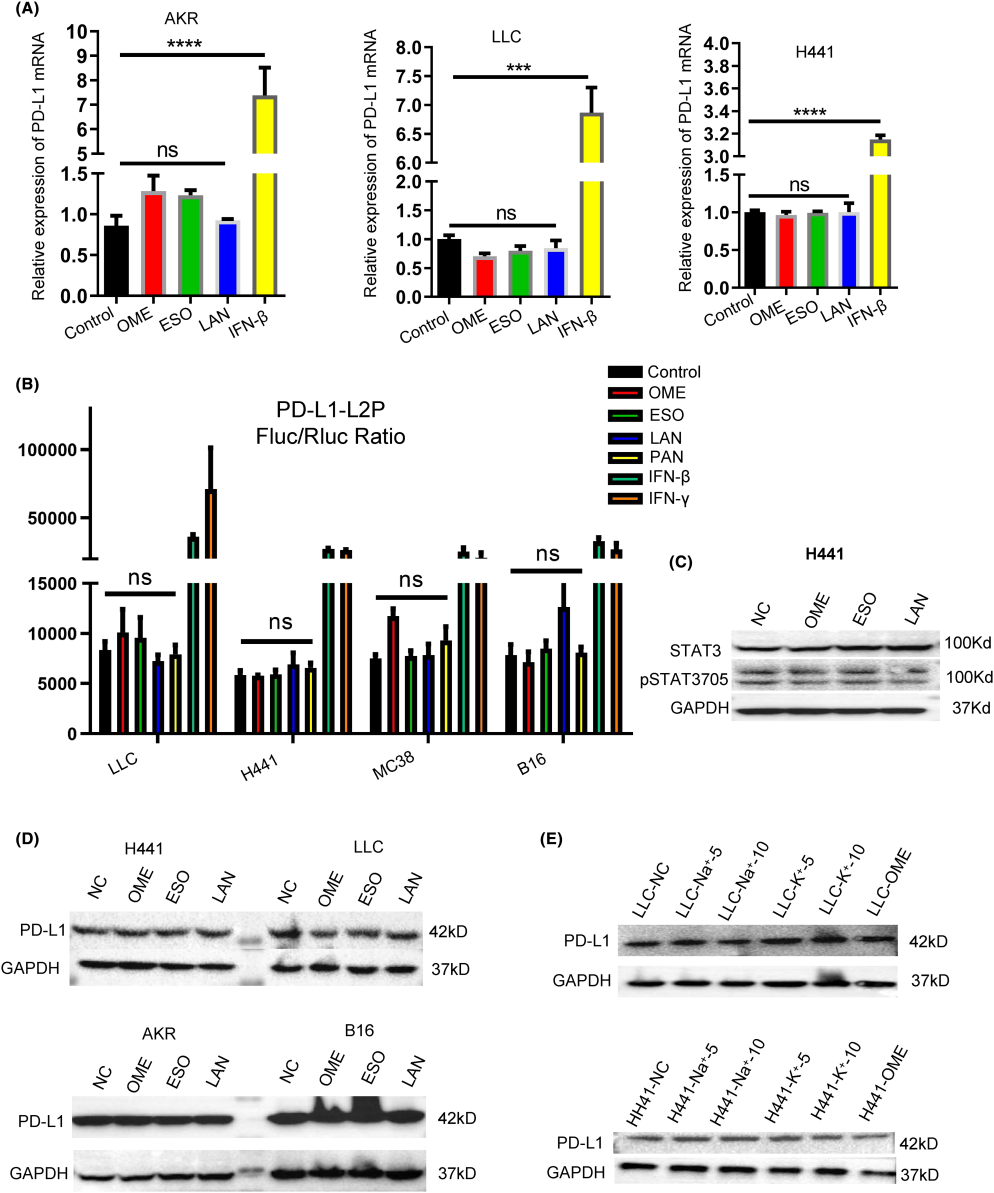
PPIs did not affect PD‐L1 mRNA transcription and total protein changes. (A) AKR, LLC and H441 cells were treated with omeprazole, esoprazole, panprazole, and IFN‐β (positive control) for 12 h before PD‐L1 mRNA was subsequently analyzed by quantitative real‐time PCR with the indicated primers. (B) HEK‐293 T cells were transfected with PD‐L1 luciferase reporter and internal control renilla luciferase reporter, followed by treatment with omeprazole, esoprazole, panprazole, and IFN‐β (positive control) for 12 h before the luciferase reporter assays were performed. (C) The pSTAT3‐705 and STAT3 protein expression in H441 cells were detected by Western blotting after treated with omeprazole, esoprazole, and panprazole for 24 h. (D) The PD‐L1 protein expression in AKR, LLC, H441, and B16 tumor cells were detected by Western blotting after treated with omeprazole, esoprazole, and panprazole for 24 h. (E) The PD‐L1 protein expression in AKR, LLC, and H441 tumor cells were detected by Western blotting after treated with Na^+^ or K^+^ for 24 h. *p ≤ 0.05, **p ≤ 0.01, ***p ≤ 0.001, ****p ≤ 0.0001.

As mentioned before, we previously demonstrated that PPIs could promote PD‐L1 translocation onto cell membrane in a variety of tumor cells, while quantitative real‐time PCR results showed that PD‐L1 mRNA levels were not significantly changed after PPIs treatment (IFN‐β or IFN‐γ were used as positive controls) (Figure 3A; Figure S4C). The double luciferase reporter assays also confirmed that administration of PPIs did not promote PD‐L1 transcription in multiply tumor cells (Figure 3B), and there were no changes in pSTAT3, a well‐known transcription factor for inducing PD‐L1 expression, observed as well (Figure 3C). Additionally, total amount of PD‐L1 protein also did not increase after PPIs treatment (Figure 3D; Figure S4B). Since PPIs could directly suppress the activation of H^+^‐K^+^‐ATPase to affect the normal cytoplastic and ectocytic ion homeostasis, changing the Na^+^ and K^+^ concentration in cell culture medium did not affect the PD‐L1 translocation as well (Figure 3E). These observations suggested that PPIs might not affect the PD‐L1 transcription.

### 3.4 PPIs promote PD‐L1 transfer onto cell membranes via GSK3β phosphorylation

**FIGURE 4:**
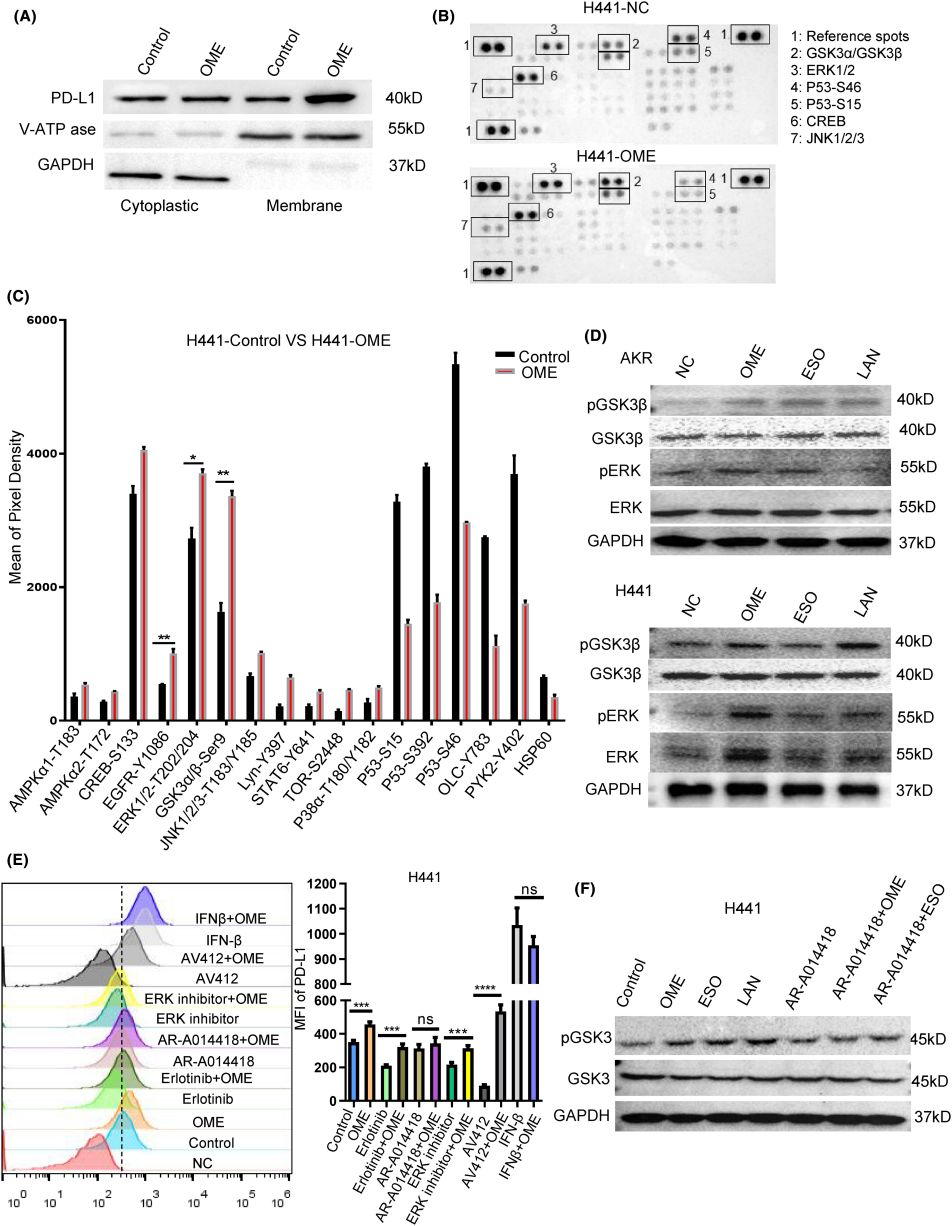
PPIs promote PD‐L1 transfer to cell membranes via GSK3β phosphorylation. (A) The PD‐L1 protein expression in H441 cytoplasm and cell membrane (purified by cellular membrane kit, No. P0033, Beyotime) was detected by western blotting after treated with omeprazole for 24 h. (B) Phospho‐antibody protein array were used to perform the PD‐L1 related signal pathway proteins in H441 cells (control vs. omeprazole for 24 h). (C) The relative changes of phospho‐antibody proteins in H441 cells were visualized by GrafPad software. (D) The PD‐L1, GSK3β, pGSK3β‐ser9, ERK, pERK202/204 protein level in AKR, and H441 tumor cells were detected by Western blotting after treated with omeprazole, esoprazole, and panprazole for 24 h. (E) FACS analysis was used to perform the MFI of cell membrane of PD‐L1 in H441 cells with control or multiply small molecular inhibitors (Seen in the Table 1, 24 h) (*n* = 3). (F) The PD‐L1, GSK3β, and pGSK3β‐ser9 protein level in H441 cells were detected by Western blotting after treated with AR‐A014418 (a small molecular inhibitors of GSK3β), omeprazole, and esoprazole for 24 h. *p ≤ 0.05, **p ≤ 0.01, ***p ≤ 0.001, ****p ≤ 0.0001.

Therefore, it was reasonable to hypothesize that PPIs might regulate PD‐L1 posttranslational modification, subsequently inducing PD‐L1 protein movement to the cell membrane. Employing membrane protein extraction kit to purify the cell membrane protein, and as respected, we observed an obviously up‐regulation of the PD‐L1 expression was obtained in Figure 4A. Based on this phenomenon, we designed a phosphorylated chip encompassing multiply PD‐L1 regulatory signaling pathways, included PD‐L1 transportation, degradation, and posttranslational modification, to further explore the potential mechanism of PPIs action on PD‐L1 expression. Analysis the differences of proteins with or without PPIs, we observed that the phosphorylated levels of GSK3β‐Ser9, EGFR‐Y1086, ERK‐Thr202/204, and CREB‐Ser133 were upregulated, while the P53 and PYK2‐Y402 was decreased in human H441 tumor cells(Figure 4B,C). Western blotting assays also indicated significant upregulation of phosphorylated GSK3β‐Ser9 and ERK‐Thr202/204 following OME and ESO treatment (Figure 4D). Combined with these results, we confirmed that PPIs might influence PD‐L1 membrane expression through GSK3β‐Ser9 or ERK‐Thr202/204. To further confirm whether the phosphorylation of EGFR, GSK3β or ERK were involved in the regulation of PD‐L1 membranic up‐regulation induced by PPIs. We pretreated H441, LLC, and B16 cells with EGFR signaling pathway inhibitors (Erlotinib and AV412), GSK3β small molecule inhibitors (AR‐A014418) and ERK1/2 small molecule inhibitors (ERK1/2 inhibitor 2), followed by PPIs treatment. We also confirmed that GSK3β inhibitors did not downregulate PD‐L1 expression, while EGFR and ERK inhibitors impair the PD‐L1 expression (seen in Figure S4D). PPIs lost their capacity of upregulation of PD‐L1 expression when GSK3β function was inhibited by the small molecule inhibitor (Figure 4E,F). These results confirmed that PPIs might promote PD‐L1 expression in tumor cell membranes through GSK3β phosphorylation.

### 3.5 PPIs promote the expression of PD‐L1 in cancer tissue samples

**FIGURE 5:**
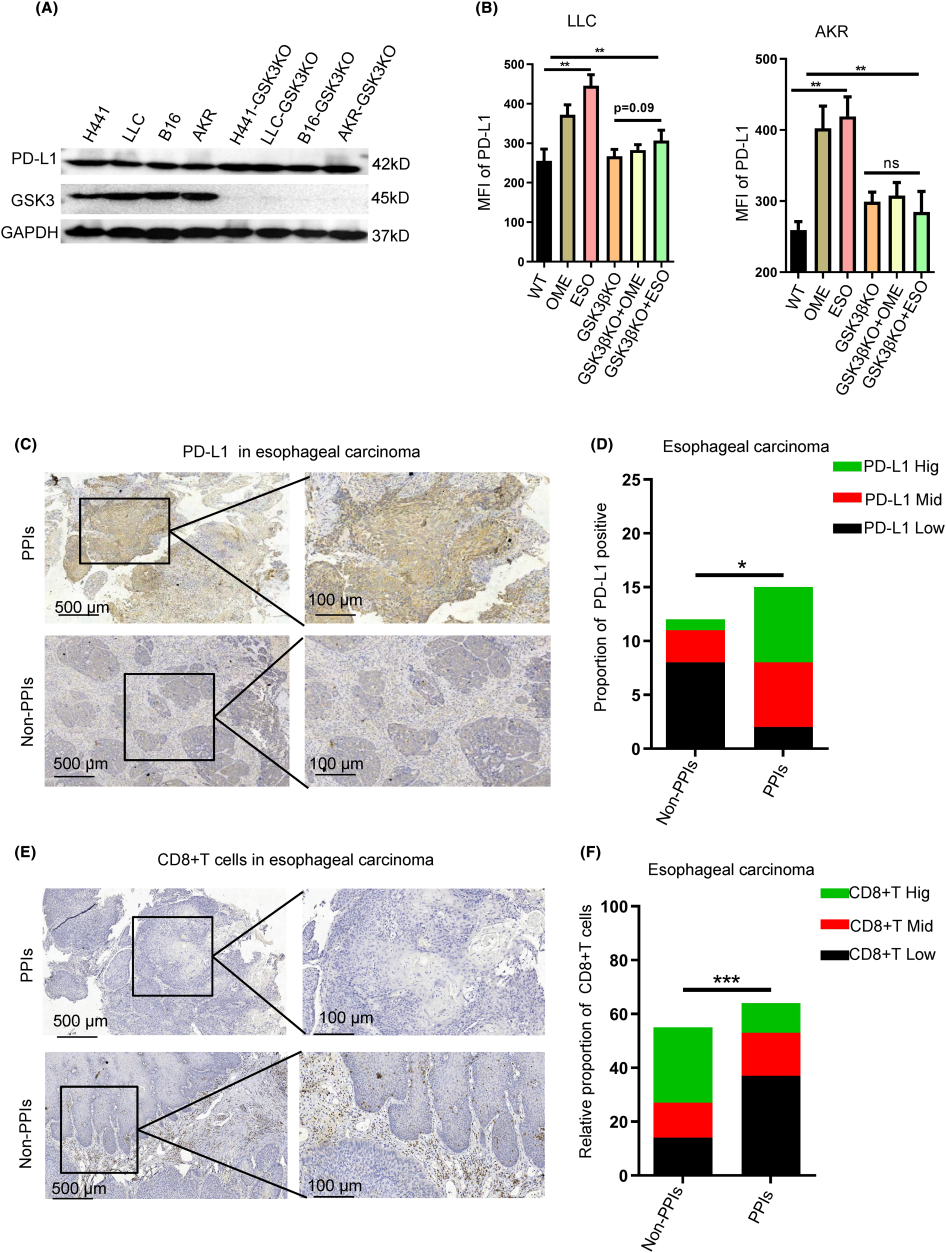
PPIs up‐regulated the expression of PD‐L1 in human tissue samples. (A) GSK3β and PD‐L1 expression were detected by western blotting in H441, B16, AKR, and LLC cells transfected with an empty control Lenti V2‐plasmid (GSK3β‐NC) or Lenti V2‐sgGSK3β‐plasmid (sgGSK3β and GSK3β KO). (B) FACS analysis was used to perform the MFI of cell membrane of PD‐L1 in control or GSK3β KO of LLC, AKR, and H441 cells after treated with omeprazole and esoprazole. *p ≤ 0.05, **p ≤ 0.01, ***p ≤ 0.001, ****p ≤ 0.0001. (C) IHC staining for PD‐L1 were performed from the esophageal carcinoma patient tissue with or without usage of PPIs (*n* = 60). Representative images of cell staining intensity were shown. (D) Statistical analysis of the expression of PD‐L1 in PPIs‐ used specimens compared with non‐PPIs used esophageal carcinoma tissue was shown. (E) IHC analysis showed the proportion of CD8T^+^ tumor infiltrating lymphocyte (TIL) cells from the esophageal carcinoma patient tissue with or without usage of PPIs (*n* = 60). Representative images of cell staining intensity were shown. (F) Statistical analysis of the expression of CD8T^+^ tumor infiltrating lymphocyte (TIL) cells in PPIs‐used specimens compared with non‐PPIs used esophageal carcinoma tissue was shown. *p ≤ 0.05, **p ≤ 0.01, ***p ≤ 0.001, ****p ≤ 0.0001.

We further knocked out GSK3β in H441, LLC, AKR, and B16 cells by CRISPR‐CAS9 technology. Subsequently results showed that PD‐L1 expression was not significantly changed after GSK3β deficiency (Figure 5A). After treating GSK3β‐KO tumor cells with PPIs and then detected the expression of PD‐L1 on tumor cell membrane, we found that OME and ESO were ineffective in promoting membranous expression of PD‐L1 (Figure 5B). To further verify the correlation between PPIs application and PD‐L1 expression in clinical samples. We initially collected pathological tissue samples from 60 clinical patients diagnosis with esophageal cancer or reflux esophagitis. According to intake PPIs or not, they were divided into control group (non PPIs group) and experimental group (PPIs group). By immunohistochemistry, we found that both in esophageal cancer and reflux esophagitis, the PD‐L1 expression in patients in taking PPIs was significantly higher than those of control group (Figure 5C,D). Moreover, a decreasing proportion of tumor‐infiltrated CD8^+^T cells was observed in PPIs intake group than that of PPIs group in esophageal cancer patients (Figure 5E,F). The similar results verified that PPI usage promoted the PD‐L1 expression in reflux esophagitis (Figure S3A,B). In conclusion, the PPIs application could promote the PD‐L1 expression in tumor tissues resulted in an immunosuppressive microenvironment.

## 4 DISCUSSION

ICIs have achieved an indelible breakthrough in the field of cancer immunotherapy.^24^ However, irAEs limits potential patient population who could benefit from ICIs immune therapy. A thorough understanding of the mechanisms underlying the development of irAEs and the implementation of appropriate strategies are crucial for enhancing the efficacy of immune checkpoint therapy.^25^ Herein, our present study offers a novel insight into a novel upregulated mechanism of PD‐L1 expression in malignant tumor cells by administrating a commonly antacid secretion drug named PPIs. Specifically, PPIs were found to promote PD‐L1 expression on the membranes of various malignant cells by inducing GSK3β phosphorylation, thereby facilitating tumor progression and resulting in an increased risk of immunosuppression.

PPIs, commonly used to suppress gastric acid relate‐diseases, regarded as having a double‐sword function in cancer progression by previously publications.^26^ Epidemiological studies have reported long‐term of PPIs intake is positively association with the higher risk of gastric, colon, and non‐smaller lung cancer cells.^12^, ^27^, ^28^ In contrast, in vitro, and in vivo studies have confirmed that PPIs also inhibit the function of V‐ATPase, which regulating intracellular pH homeostasis for maintaining tumor cells survival, inducing the mitochondrial apoptosis and impairing tumor growth.^29^, ^30^ Elevated expression of V‐ATPase is significantly associated with poor survival in variety of cancers.^31^ Pre‐treatment with OME and ESO enhanced the efficiency of various anticancer agents (CDDP, 5‐FU, and vinblastine) via inhibition of V‐ATPase enzymatic activity in human melanoma, adenocarcinoma and lymphoma.^32^ However, Ana Babic et al. argue the opinion that use of PPIs or H2RA was not associated with higher risk of colorectal cancer.^33^ Further studies also suggest that elevated cancer risks are primarily restricted to those with over 10 years of PPI use, with no consistent associations found for increasing PPI dose and/or duration of use.^34^ We confirmed that the IC_50_ of PPIs in fibroblasts was significantly higher than tumor cell lines, indicating that PPIs are less toxic than normal epithelial cells. And low concentrations of PPIs did not affect tumor proliferation. Therefore, the effects of PPIs on solid malignancies' response to ICIs warrant further exploration.

Prior studies have suggested that PPIs can affect the acidic TME and intestinal flora, thereby indirectly hindering the efficacy of ICIs.^35^, ^36^ PPIs usage resulted in a significant reduction of the intestinal microbic diversity, specifically inducing to a decrease of *Bifidobacterium*, Rumen Coccidiaceae, Aniscospiraceae, and *Moliaceae*, but an increase of *Clostridium difficile* and *Enterobacteriaceae*, ultimately led to a significant inhibition of the ICIs response.^14^, ^37^ Till date, whether PPIs could directly regulate the expression of immune checkpoints such as PD‐L1 or PD‐1 has not been reported. Here, we identified that PPIs increase the stability of PD‐L1 protein by inducing GSK3β phosphorylation. These findings aligns with another publication that suggests GSK3β stabilizes PD‐L1 protein expression by upregulating glycosylation.^38^ In addition, GSK3β may inhibit PD‐L1 degradation by inhibiting β‐TrCP‐ubiquitination signaling.^39^ We also obtain that PPIs synergize with PD‐L1 antibodies to inhibit tumor progression in xenograft mouse models. Moreover, our clinical data confirm that PPIs are positively correlated with the protein levels of PD‐L1 in esophageal cancer, suggesting that usage of PPIs might play a significant role in promoting tumor PD‐L1 expression. Our study still have some limitations. It is known that GSK3β phosphorylation promotes PD‐L1 protein expression through phosphorylation and glycosylation modifications, the specific type of posttranslational modifications induced by PPIs on PD‐L1 requires further investigation. Additionally, the limited scale and diversity of clinical samples in this study lead to some uncertainty in translating these findings to a clinical setting.^14^, ^37^

In summary, we report a significant mechanism whereby PPI usage directly induces PD‐L1 expression by stimulating GSK3β phosphorylation, thus facilitating primary tumor progression and metastasis.

## AUTHOR CONTRIBUTIONS

**Long Gao:** Funding acquisition (supporting); methodology (lead). **Yuan Liu:** Data curation (supporting); methodology (supporting). **Jiaying Liu:** Conceptualization (supporting); project administration (supporting). **Jiali Li:** Conceptualization (supporting); formal analysis (supporting). **Haotian Li:** Investigation (supporting); methodology (supporting). **Yanyan Liu:** Data curation (supporting); funding acquisition (supporting). **Fang Meng:** Conceptualization (supporting); project administration (supporting). **Xiaohong Du:** Conceptualization (lead); data curation (supporting); investigation (supporting). **Yufeng Gao:** Conceptualization (lead); funding acquisition (supporting); methodology (supporting). **jiabin li:** Funding acquisition (lead); investigation (lead). **F. Xiao‐Feng Qin:** Conceptualization (lead); investigation (lead).

## FUNDING INFORMATION

This work was supported by: the National Natural Science Foundation of China (Nos. 81973983, 82270015, 82100017, 82302577, 82304209), Anhui Province scientific research planning project (2023AH010083, 2023AH053282), the joint construction project of clinical medicine university and hospital (No. 2021lcxk006), the Natural Science Foundation in Anhui Province (Nos. 2208085MH264, 2308085QH284, 2308085MH243), China Primary Health Care Foundation (No. MTP2022A015) and the Project Supported by Anhui Medical University (2021xkj138), Post‐doctoral scientific research project of Anhui Province (No. 2022B609).

## CONFLICT OF INTEREST STATEMENT

The authors declare competitive conflicts of interest. The authors apologize to the scientists whose work was not cited because of space limitations.

## ETHICS STATEMENT

All animal experiments were agreed with animal care, handling, and treatment according to the guidelines approved by the Institutional Animal Care and Use Committee of Suzhou Institute of Systems Medicine (ISM‐IACUC‐0003‐R).

## CONSENT FOR PUBLICATION

We confirm that the manuscript has not been published elsewhere. All authors have contributed significantly to the work, and have read and agreed on the content of the final version of the manuscript.

## Supporting information


**Figure S1.** PPIs significantly up‐regulated the PD‐L1 membrane expression in tumor cells. (A) Representative FACS plots showing the relative PD‐L1 expression in LLC and H441 cells treated with different type of PPIs (*n* = 3). (B) Representative FACS plots showing the relative PD‐L1 expression in AKR cells treated with the different concentration of Esomeprazole for 24 h (ESO) (*n* = 3). *p ≤ 0.05, **p ≤ 0.01, ***p ≤ 0.001, ****p ≤ 0.0001.

**Figure S2.** PPIs synergistic enhanced the efficacy of PD‐L1 antibody. (A) LLC and LLC‐T2 cells were separately implanted into C57BL/6 mouse models (2 × 10^5^ cells per mouse). Tumors were measured every 2 days at Day 7. The tumor growth curve was shown with tumor sizes (*n* = 5). (B) LLC cells (2 × 10^5^ per mice) were separately implanted into C57BL/6 mouse (left). Tumors were measured every 2 days at Day 7, and then Esoprazole (20 mg/kg), PD‐L1 antibody (10 mg/kg), the combination of the above drugs, or PBS control was injected into mice (intraperitoneal) twice a week for 2 weeks beginning on the ninth day after LLC tumor cells were subcutaneously implanted (left). The tumor growth curve was shown with tumor sizes (*n* = 5–6). Mice were sacrificed at Day 18 after injection. The primary tumor mass is shown on the right (right). (C) LLC‐T2 cells (2 × 10^5^ per mice) were separately injected into tail vein of C57BL/6 mouse (left), and then Esoprazole (20 mg/kg), PD‐L1 antibody (10 mg/kg), the combination of the above drugs, or PBS control was injected into mice (intraperitoneal) twice a week for 2 weeks beginning on the ninth day after LLC tumor cells injected. Number of spontaneous lung metastases in LLC‐T2 tumor‐bearing immunocompetent C57BL/6 mouse was shown. The visual numbers were randomly chosen in each group (*n* = 3), calculated by image J software. *p ≤ 0.05, **p ≤ 0.01, ***p ≤ 0.001, ****p ≤ 0.0001.

**Figure S3** PPIs up‐regulated the expression of PD‐L1 in human tissue samples (A) IHC staining for PD‐L1 were performed from the Reflux esophagitis patient tissue with or without usage of PPIs (*n* = 60). Representative images of cell staining intensity were shown. (B) Statistical analysis of the expression of PD‐L1 in PPIs‐ used Reflux esophagitis specimens compared with non‐PPIs used tissue was shown.

**Figure S4.** PPIs promoted PD‐L1 tranfer to cell membrane. (A) Fluorescence microscopy assessed the PD‐L1 expression in H441 cell lines treated with OME for 24 h. (B) The PD‐L1 protein expression in H441 cells were detected by western blotting after treated with Omeprazole and Esoprazole for 24 h and 48 h. (C) The PD‐L1 mRNA in LLC cells were detected by qRT‐PCR after treated with Omeprazole, IFNβ (10 ng/mL only for 24 h) and Esoprazole for 24 h and 48 h. (D) The PD‐L1 protein expression in LLC cells were detected by Western blotting after treated with Omeprazole, AR‐A014418, erlotinib, and ERK1/2 inhibitor for 24 h. *p ≤ 0.05, **p ≤ 0.01, ***p ≤ 0.001, ****p ≤ 0.0001.

## Data Availability

Specific details will be provided if request.
