# Supplementary figures and images for: Proton pump inhibitors stabilize the expression of PD‐L1 on cell membrane depending on the phosphorylation of GSK3β
Source: Cancer Med. 2024 May 16;13(10):e7083. doi: 10.1002/cam4.7083 (PMC11097254; doi:10.1002/cam4.7083)

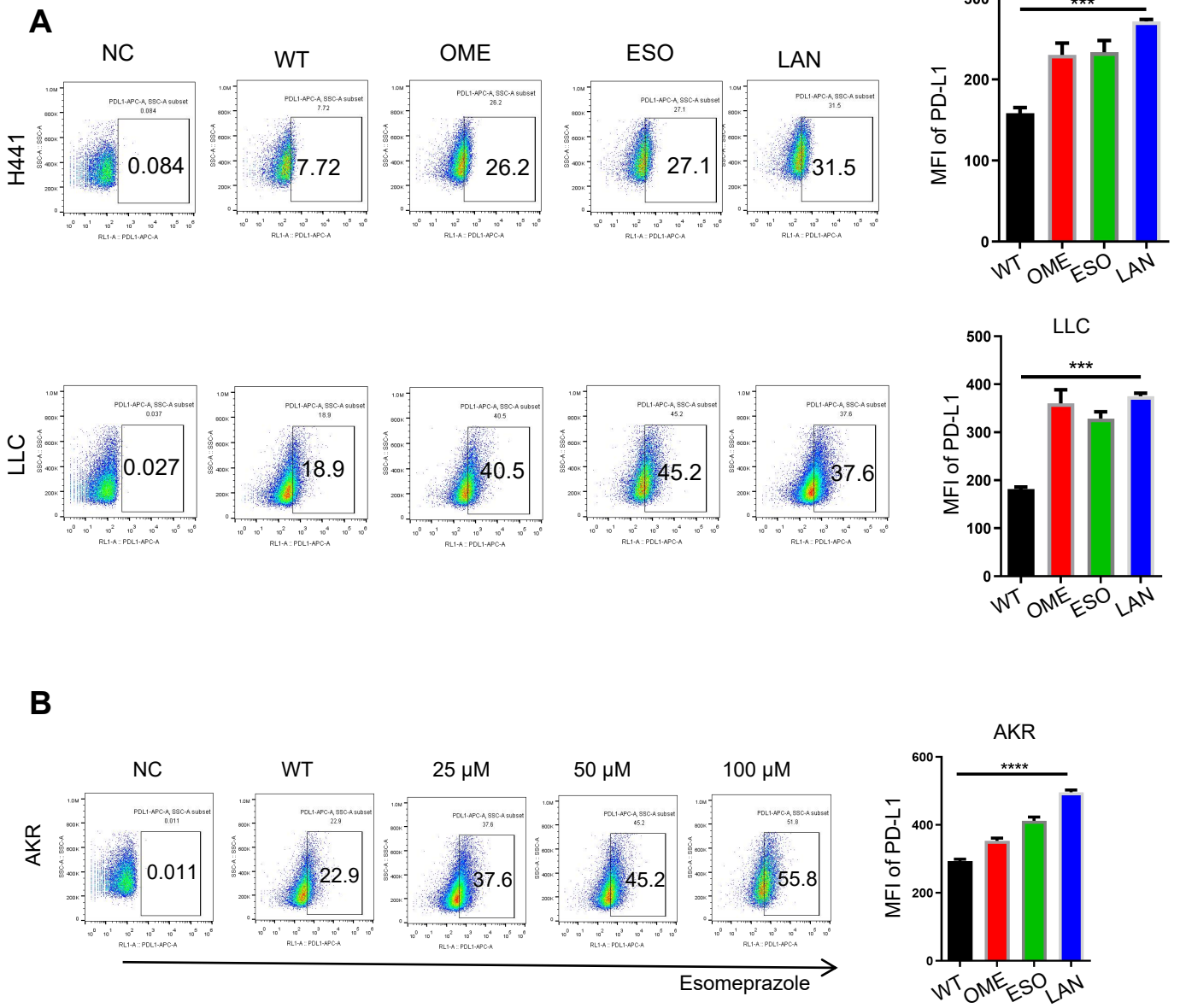

Supplement: Supplementary file 1 — Figure S1. PPIs significantly up‐regulated the PD‐L1 membrane expression in tumor cells. (A) Representative FACS plots showing the relative PD‐L1 expression in LLC and H441 cells treated with different type of PPIs (n = 3). (B) Representative FACS plots showing the relative PD‐L1 expression in AKR cells treated with the different concentration of Esomeprazole for 24 h (ESO) (n = 3). *p ≤ 0.05, **p ≤ 0.01, ***p ≤ 0.001, ****p ≤ 0.0001. [file CAM4-13-e7083-s002.pdf]

**A**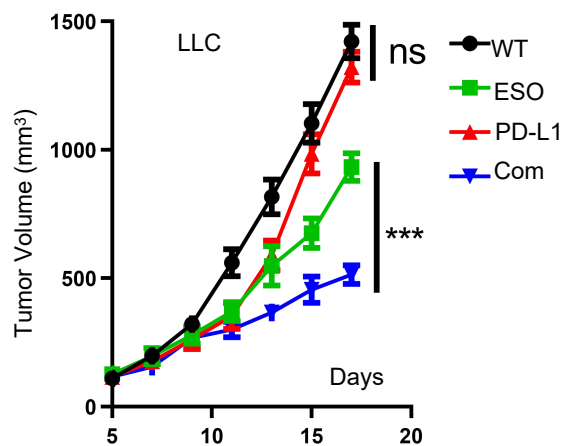**B**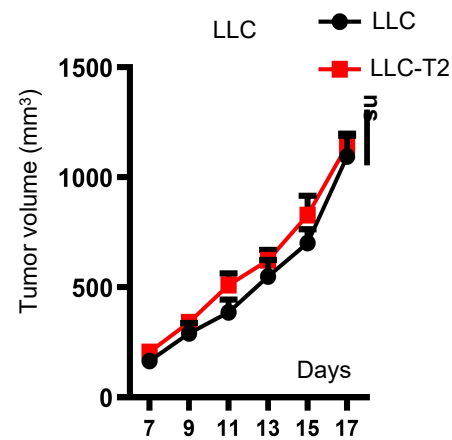**C**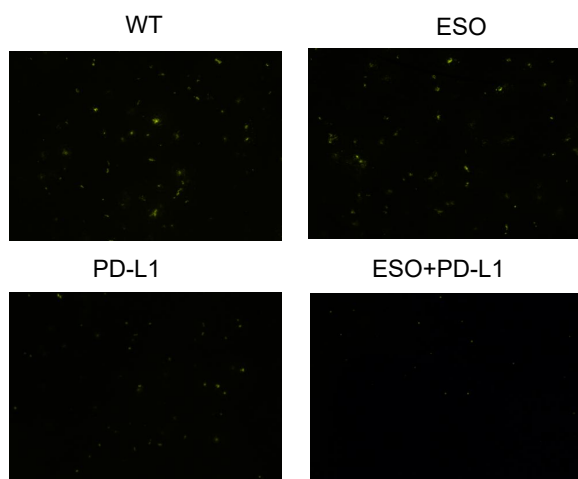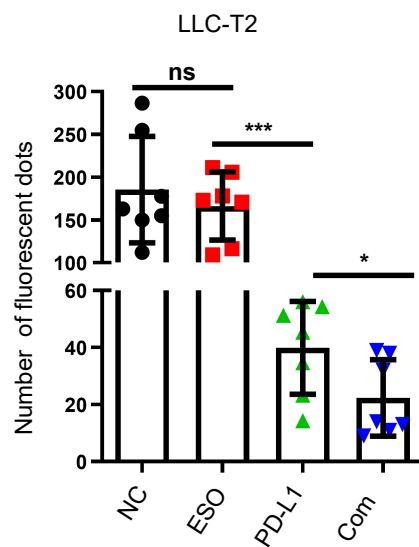

Supplement: Supplementary file 2 — Figure S2. PPIs synergistic enhanced the efficacy of PD‐L1 antibody. (A) LLC and LLC‐T2 cells were separately implanted into C57BL/6 mouse models (2 × 10^5^ cells per mouse). Tumors were measured every 2 days at Day 7. The tumor growth curve was shown with tumor sizes (n = 5). (B) LLC cells (2 × 10^5^ per mice) were separately implanted into C57BL/6 mouse (left). Tumors were measured every 2 days at Day 7, and then Esoprazole (20 mg/kg), PD‐L1 antibody (10 mg/kg), the combination of the above drugs, or PBS control was injected into mice (intraperitoneal) twice a week for 2 weeks beginning on the ninth day after LLC tumor cells were subcutaneously implanted (left). The tumor growth curve was shown with tumor sizes (n = 5–6). Mice were sacrificed at Day 18 after injection. The primary tumor mass is shown on the right (right). (C) LLC‐T2 cells (2 × 10^5^ per mice) were separately injected into tail vein of C57BL/6 mouse (left), and then Esoprazole (20 mg/kg), PD‐L1 antibody (10 mg/kg), the combination of the above drugs, or PBS control was injected into mice (intraperitoneal) twice a week for 2 weeks beginning on the ninth day after LLC tumor cells injected. Number of spontaneous lung metastases in LLC‐T2 tumor‐bearing immunocompetent C57BL/6 mouse was shown. The visual numbers were randomly chosen in each group (n = 3), calculated by image J software. *p ≤ 0.05, **p ≤ 0.01, ***p ≤ 0.001, ****p ≤ 0.0001. [file CAM4-13-e7083-s003.pdf]

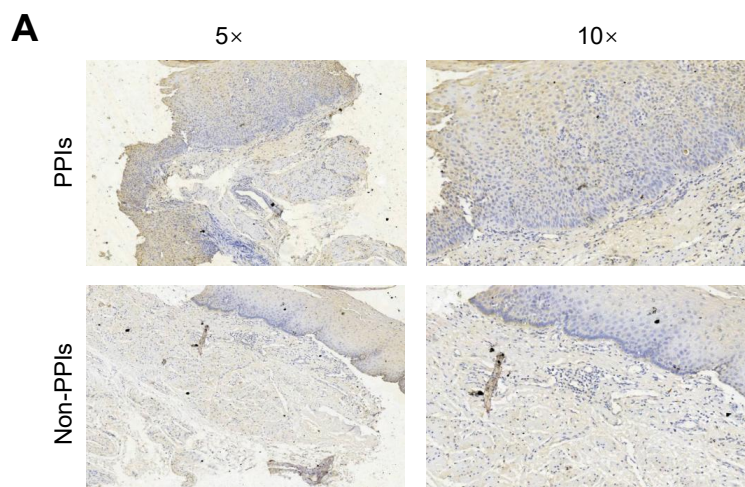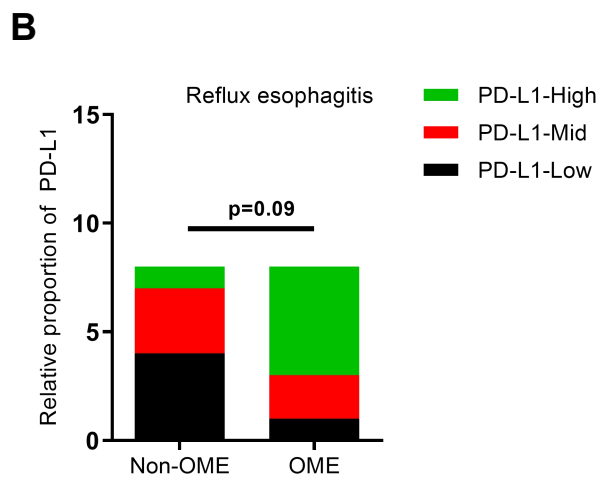

Supplement: Supplementary file 3 — Figure S3 PPIs up‐regulated the expression of PD‐L1 in human tissue samples (A) IHC staining for PD‐L1 were performed from the Reflux esophagitis patient tissue with or without usage of PPIs (n = 60). Representative images of cell staining intensity were shown. (B) Statistical analysis of the expression of PD‐L1 in PPIs‐ used Reflux esophagitis specimens compared with non‐PPIs used tissue was shown. [file CAM4-13-e7083-s004.pdf]

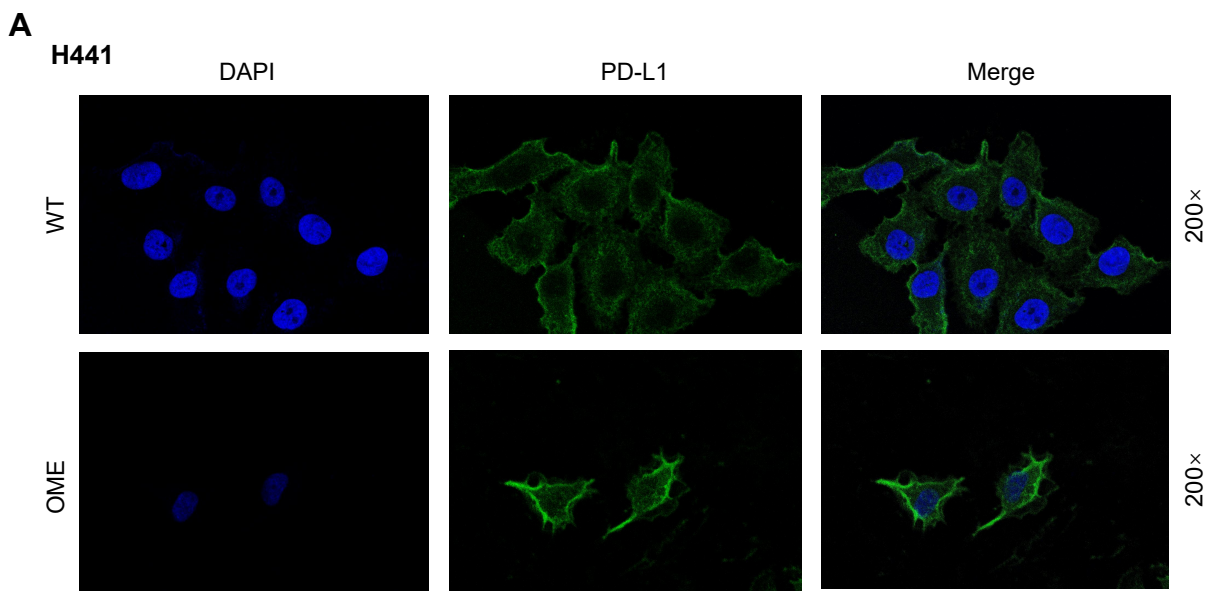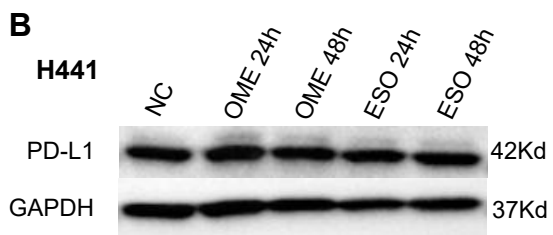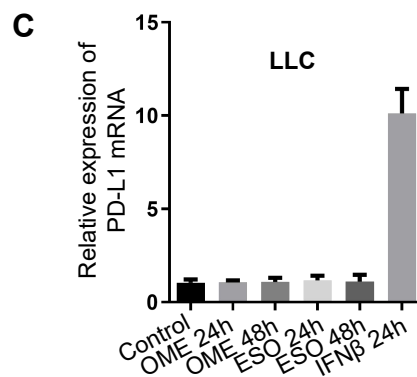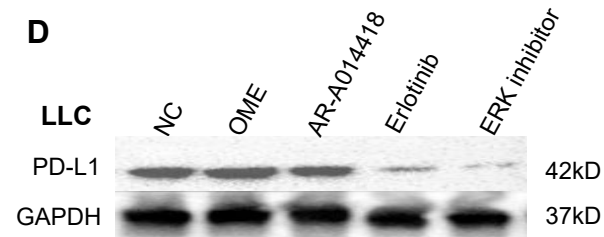

Supplement: Supplementary file 4 — Figure S4. PPIs promoted PD‐L1 tranfer to cell membrane. (A) Fluorescence microscopy assessed the PD‐L1 expression in H441 cell lines treated with OME for 24 h. (B) The PD‐L1 protein expression in H441 cells were detected by western blotting after treated with Omeprazole and Esoprazole for 24 h and 48 h. (C) The PD‐L1 mRNA in LLC cells were detected by qRT‐PCR after treated with Omeprazole, IFNβ (10 ng/mL only for 24 h) and Esoprazole for 24 h and 48 h. (D) The PD‐L1 protein expression in LLC cells were detected by Western blotting after treated with Omeprazole, AR‐A014418, erlotinib, and ERK1/2 inhibitor for 24 h. *p ≤ 0.05, **p ≤ 0.01, ***p ≤ 0.001, ****p ≤ 0.0001. [file CAM4-13-e7083-s001.pdf]
